# Bioactive Betalain Extracts from Cactus Pear Fruit Pulp, Beetroot Tubers, and Amaranth Leaves

**DOI:** 10.3390/molecules26165012

**Published:** 2021-08-18

**Authors:** Vuyisa Sigwela, Maryna De Wit, Alba du Toit, Gernot Osthoff, Arno Hugo

**Affiliations:** 1 Department of Sustainable Food Systems and Development, University of the Free State, P.O. Box 339, Bloemfontein 9301, South Africa; SigwelaVN@ufs.ac.za (V.S.); dutoita1@ufs.ac.za (A.d.T.); 2 Department of Microbial, Biochemical and Food Biotechnology, University of the Free State, P.O. Box 339, Bloemfontein 9301, South Africa; OsthoffG@ufs.ac.za; 3 Department of Animal Science, University of the Free State, P.O. Box 339, Bloemfontein 9301, South Africa; HugoA@ufs.ac.za

**Keywords:** amaranth, antioxidants, ascorbic acid, beetroot, betacyanins, cactus pear, flavonoids, indicaxanthan, polyphenols

## Abstract

Natural food items and the additional benefits they provide have received considerable attention in recent years. Betalains are nutritious pigments which have valuable biological properties, e.g., antioxidant and free radical scavenging activity. In this study, aqueous betalain extracts were obtained from different coloured cactus pears (purple, red/pink, and orange), amaranth, and beetroot, with and without the addition of ascorbic acid, microwave-heated, and freeze-dried and subsequently analysed by thin layer chromatography (TLC). Beetroot samples without the addition of ascorbic acid (AA) had lower phenols, flavonoids, and ascorbic acid content than beetroot samples extracted with the addition of AA. Amaranth had significantly higher contents of antioxidants than all the other plants. Results for phenolic compounds showed that there were no significant differences between cactus pear cultivars, however, significant differences were seen between the two beetroot samples (microwave-heated with and without AA) as well as amaranth. For flavonoid compounds, amaranth had significantly higher values than all other samples. The lowest flavonoid content was found in beetroot without AA (0.49 mgCE/g). For ascorbic acid, significant differences were noticed between amaranth (71.71 mg/100 g) and samples from cactus pear and beetroot. TLC results showed that purple and red cactus pear samples had the most vivid colours, a reflection of the high betacyanin and betaxanthin contents in the cultivars. Moreover, extracts from cactus pear, beetroot, and amaranth were classified according to a decision tree which was designed by the Code of Federal Regulations/Food Additives Regulation of the EU. The classification of betalain pigment extracts as colouring foods was achieved through enrichment factor calculations and the colourant decision tree. The results showed that the betalain pigment extraction method used is inexpensive, time-saving, energy-saving, non-toxic, and chemical solvent free and yields high concentrations of betalains.

## 1. Introduction

Betalains are nutrient-dense pigments that have both colouring and antioxidant properties. These pigments are regarded as functional ingredients for foods which have anti-lipidemic and anti-cancer properties [1]. The healing properties contribute to preventing non-communicable diseases such as diabetes, hypertension, cardiovascular disease, and obesity [2,3]. These beneficial attributes stem from their high micronutrient presence of flavonoids and ascorbic acid, amongst other nutrients [4,5]. Several health-related biological functions (properties) have been assigned to betalain rich foods, i.e., anti-inflammatory, antimicrobial, lipid peroxidation prevention, DNA-damage prevention, gene regulation, anti-proliferative and free radical scavenging [6], causing supplementation with betalain rich foods to treat inflammation (intestinal), cancer, oxidative stress-related and dyslipidemia diseases such as hypertension and atherosclerosis, cardiovascular diseases, diabetes, and asthma [7].

Only a few fruits and vegetables contain betalains and the best known is beetroot (*Beta vulgaris*) that produces betanin, an important food colourant. Other edible sources are *Opuntia* spp. fruits (prickly and cactus pear), *Hylocerasus* cacti (e.g., *Hylocereus polyrhizus*), and grains or leaves of amaranth (*Amaranthus* spp.) [8]. Betalains are found in the vacuoles as dissolved *bis*-ions. ‘Betalains’ are divided into two main structural groups: the red to red-violet betacyanins (Bc) and the yellow-orange betaxanthins (Bx). With new methods and technologies, 180 different betacyanin pigments have already been described [9]. These are secondary metabolites that derive from l-tyrosine (polar aromatic amino acid) via the formation of l-dihydroxyphenylalanine (l-DOPA). The cleavage of l-DOPA, followed by the incorporation of a nitrogen atom into the ring forms betalate, which is common to all betalains. The yellow Bx are obtained by condensation of betalamic acid with amines and amino acids, while Bc results from their condensation with free l-DOPA derivatives. Betalains are more stable to pH and temperature than anthocyanins. However, red beetroot colour is unstable, especially in moisture/aqueous environments [8]. Betalain stability is influenced by internal factors such as pH, moisture content, pigment content, chemical structure and food matrix, as well as external factors such as temperature, light, and oxygen [10], pH, water activity, enzymes, and food additives (antioxidants and chelating agents), as well as metal cations [7]. Betacyanins and betaxanthins show different sensitivities towards treatments, especially heat treatment. Possible changes after processing and storage of betalains include deglycosylation, isomerization, hydrolysis, decarboxylation, hydrogenation, and the breakdown of the aldimine bond [7].

Betalains are usually extracted from ground material (plant sources) by maceration with water, ethanol, or methanol, sometimes acidified, either cold or at room temperature or by Soxhlet extraction. More environmentally friendly extraction methods such as diffusion, ultrafiltration, reverse osmosis, pulsed electric fields, gamma irradiation, microwave-assisted, ultrasound-assisted, aqueous two-phase extraction, and cryogenic freezing have been developed and used. Microwave-assisted extraction (MAE) includes the use of microwave energy to heat solvents containing active compounds by partitioning analytes from the sample matrix/matrices into the solvent [11,12]. Microwave energy are electromagnetic fields of frequencies between 300 MHz and 300 GHz, comprising of two oscillating perpendicular fields (a magnetic and an electric field) [13]. Microwaves increase the kinetics of the reaction by transformation of kinetic energy to thermal energy. MAE is regarded as a non-conventional, relevant, novel, innovative, clean, and green extraction process used for the extraction of organic and organometallic compounds, such as antioxidants, pigments, spices, and oil from various matrices. Active compounds such as terpenes, alkaloids, flavones, glycosides, and essential oils are extracted from medicinal plants, herbs, etc. It is considered as one of the best extraction methods for pigments like betalains, anthocyanins, paprika, and carotenoids. Its advantages include and combine rapidity, reproducibility, high extraction yields, lower energy input, toxic-free mediums, less solvents, reduced sample size and equipment size, selectivity, protection against chemical transformation, higher temperatures without damaging the pigment, homogeneous heating as well as better qualities and quantities of the desired products (efficiency) [11,12,13,14,15,16]. Since betalains are heat-sensitive, a heat-treatment with lesser exposure to heat is optimal. It was found that five minutes of microwave irradiation produced the same yields of phenolic compounds and flavonoids than treatment with ultrasonic waves for one hour [17]. MAE is regarded as a clean process since it induces frustule permabilization but avoids frustule explosion. It is a relevant and innovative technique, using higher temperatures without damaging the pigment [14].

The cactus pear, amaranth, and beetroot plants are good sources of ascorbic acid, antioxidants, and phenolics. Betalains are obtainable from fruit (cactus pear) and vegetables (beetroot and amaranth) and are classified as colouring foods [18,19]. Colouring foods are foods with colouring properties which are extracted with the primary intention to add colour to food products [18].

This manuscript reports on the analysis and comparison of the betalain extract properties (antioxidant compounds) from six cactus pear cultivars, beetroot, and amaranth watery extracts. A microwave-assisted extraction method, that is chemical solvent free, low energy demand, fast and effective (elevated extraction rate) with high betalain pigment yields, was used to obtain natural food extracts which were classified according to international food laws.

Literature on the comparison of betalains from various sources are limited. The current study not only provides information on the betalain contents of different plant species, different plant parts and different fruit colours, but also provides information on its antioxidant compounds. At the same time, an attempt was made in its classification as colouring food. With this study, a novel, green, clean, and innovative extraction method was used, using water as a non-toxic, polar, and economic solvent. A relatively wide range of analytical methods (traditional and advanced) were used to provide detailed information on the aqueous betalain extracts. This study could therefore serve as a fingerprint and benchmark for future betalain characterization studies. 

## 2. Results and Discussion

### 2.1. Antioxidants

The phenolic, flavonoid, and ascorbic acid contents of beetroot extracts (microwave-heated with and without AA), amaranth extracts and cactus pear extracts (*Ficus indica*, orange; Gymno carpo, orange; Algerian, red/pink; Meyers, red/pink; Monterey, purple and Robusta, purple) is indicated in Table 1. Amaranth generally had the highest levels of antioxidants with 71.71 mg/100 g for ascorbic acid, 5.07 mg/100 g catechin equivalents (CE) flavonoids, and 34.91 mg gallic acid equivalents (GAE)/g phenols. The lowest values for ascorbic acid (11.10 mg/100 g) was found in Gymno carpo, while low values of 0.49 mg/100 g CE flavonoids, as well as 3.18 mg GAE/g phenols, were found in beetroot without AA.

During the preliminary study (mentioned and discussed in the Materials and Methods section) [20,21] the antioxidant content (flavonoids) and antioxidant capacity (DPPH reduction) of beetroot and three different coloured cactus pears (purple, red/pink, and orange) were determined by means of TLC with the addition of DPPH and FeCl_3_, respectively. DPPH is usually used and applied to do an initial screening of antioxidants based on the two main mechanisms of antioxidant action, namely single electron transfer and hydrogen atom transfer. The highest antioxidant activity was found in beetroot and purple Robusta cactus pear, while beetroot, purple Robusta and orange Gymno Carpo had similar flavonoid contents. Interestingly, Smeriglio et al. [22] found the highest ORAC values (oxygen scavenging activity) in orange cactus pear fruit. It was reported by Skalicky et al. [9] that the red pigment constituent showed a better correlation with antioxidant activity. Koss-Mikolajzyk et al. [23] reported that the antioxidant activity of betalains decreased in the following order: orange Opuntia > red Opuntia > yellow Opuntia. Antioxidant activity depends on the origin of the pigments, meaning plants synthesize different betacyanin metabolites, and these are genetically fixed [22]. Synergy between betalain classes in terms of antioxidant potential and activity should also be kept in mind.

In terms of phenol content, four groups could be identified in terms of the statistical differences: highest content in amaranth, followed by beetroot with AA, cactus pears, and the lowest content in beetroot without AA. Total phenol results showed that the beetroot samples with AA (29.86 mg GAE/g) and amaranth (34.91 mg GAE/g) were significantly higher than other samples (cactus pears). However, beetroot samples without AA (3.18 mg CE/g) were significantly lower than other samples. There was no significant difference between samples from the orange, pink/red and purple cactus pear cultivars. It was found that in Opuntia spp. that a combination of high concentrations total phenolics and Bc showed a higher antioxidant activity than lower phenolics concentration and high Bx content [24]. It was reported by Nowak et al. [25] that ascorbic acid protects polyphenol compounds, which explains the higher contents. AA is also an enhancer of phenolic extraction. It is also mentioned in the Results and Discussion and Materials and Methods sections that AA prevent quick degradation of betalain pigments and cause enhanced extraction of the pigments.

In general, vitamin C rich juices are good sources of polyphenols. Vitamin C and polyphenols act synergistically and define the antioxidant properties of juices. Furthermore, significant differences in phenolics composition are usually consistent with observed differences in antioxidant activities, since the Folin–Ciocalteu method is based on the oxidation/reduction reactions to assess the antioxidant activity [24].

Flavonoid content showed a similar pattern as was observed for phenols. Lowest values were found in the beetroot without AA, with higher values observed in the cactus pears samples, except for orange Gymno carpo (which was higher than the beetroot with AA) and the highest value found in amaranth. The highest flavonoid levels were generally found in red/purple cactus pear cultivars and Gymno carpo from the orange cultivar. These results correspond to that found in the preliminary study where the flavonoid contents were tested with TLC. Flavonoid levels of the orange cultivars Ficus indica and Gymno carpo differed significantly, although Ficus indica showed similar trends to Gymno carpo and beetroot heated with AA. All the other samples differed significantly. The highest flavonoid numerical values were found in amaranth (5.07 mg CE/g), and lowest in beetroot without AA (0.49 mg CE/g).

Ascorbic acid analysis showed a different trend than what was observed for the contents of phenols and flavonoids. There was a slight difference between the two beetroot samples, with no significant difference between the cactus pears and beetroot, while amaranth had the highest contents. The highest ascorbic acid levels were found in amaranth (71.71 mg/100 g). It was followed by the orange Ficus indica (12.35 mg/100 g), which was significantly higher than the orange Gymno Carpo (11.10 mg/100 g). Ascorbic acid is a secondary antioxidant, scavenging radicals before they enter into chain reactions [26]. In the case of apple juice, addition of ascorbic acid is usually done to stabilise Bc molecules by a slightly acidic pH and to inhibit oxidation of Bc by polyphenoloxidase and tyronase activity when Bx is present [26]. In general, acidification of betalain extracts (citric acid, acidic ethanol, etc.) improves Bc stability and avoids oxidation by PPO. Both ascorbic acid and isoascorbic acid improves betalain stability. 

The addition of specific food stabilizers (e.g., EDTA, ascorbic acid, citric acid) as well as the natural matrix compounds may exhibit stabilizing effects on the maintenance of pigments. Spectrophotometric analyses of betalains from purple pitaya fruit enabled the monitoring of reactions (betalains) during the heating of solutions (extraction of betalains at higher temperature in the presence of ascorbic acid) [27]. According to these authors, the hypsochromic shift of λmax to 460 nm is observed in more acidic media without ascorbic acid, suggesting the generation of new reaction products. The absorption bands, in solutions with ascorbic acid, are not detected, indicating the retention of the basic structure of the betalain chromophore. In general, pigment retention is diminishing with prolonged heating and it depends on the pH-values of tested solutions. The addition of ascorbic acid significantly affects the betalain stability. Bc in purple pitaya juice exhibit better heat stability, when stabilized with ascorbic acid. Therefore, a protective effect of natural juice matrix and ascorbic acid is more significant at acidic pH. 

The small difference between the ascorbic acid values of the two beetroot samples (with and without addition of ascorbic acid) could be explained by the addition of 5 mL ascorbic acid, diluting the sample volume, and thereby decreasing the content, or by the regeneration of Bc at a higher pH (the sample without ascorbic acid) or by the pro-oxidant effects of ascorbic acid or a combination of all these factors.

Du Toit [28] reported that the colour of the cactus pear fruit has an impact on the specific antioxidant content. This was further elaborated on in Du Toit et al. [29], that the highest antioxidant potential was found in purple *O. robusta* cultivar and orange *O. ficus-indica* cultivars. The ascorbic acid values reported in Du Toit et al. [29] were higher than the values reported here. In this study, the colour of pigments from cactus pear did not have an impact on antioxidant content results. 

According to Ravichandran et al. [30], extracts of beetroot that have been microwave heated or boiled are high in antioxidants. Those findings, to a certain degree, are similar to that of the current study, as antioxidants were very high in heated beetroot samples when AA was added, although the addition of AA during the extraction was already explained in the Material and Methods section [26]. Since the main objective of this study was the use of the extraction method yielding the highest betalain pigment concentration and to be used as colouring food (thus not fractionated and isolated) with an optimal red colour and without the addition of any chemicals, the extraction of betalains with the addition of AA will not be applied in future studies. 

Amaranth samples contained significantly higher levels of antioxidants compared to all other samples for total phenols, total flavonoids, and ascorbic acid. 

It has been reported that colour, determined by betalain content and properties, is a more relevant factor for prediction of the yield, stability, and food application than the source—i.e., beetroot or cactus pear. Prieto-Santiago et al. [6] used colour measurements as a quick indicator of potential health-promoting properties. According to these authors, the relationship between colour and pigment content depend on the type of matrix and pigment, as well as the factors affecting their interaction. These authors found a negative correlation between the total betalain content and the colour parameters L*, a*, b*, chroma and hue angle. Both a* and chroma was proposed as best descriptors for betalain concentrations. L/ab and a/b showed positive correlations with total betalains. As was seen in the preliminary study (results not shown) and current study, higher betalain concentrations were found in the purple tissues (amaranth, beetroot, and purple cactus pears). Changes (lower values) in L* and a* Cielab values were noticed when beetroot was extracted with ascorbic acid (results not shown). A possible explanation could be the increased Bx formation due to the condensation of free amino acids with betalamic acid generated by Bc hydrolysis, as was proposed by Herbach et al. [10]. Betalains may undergo changes after processing such as breakdown of the aldimine bond, hydrogenation, decarboxylation, isomerization, hydrolysis, and deglycosylation. These changes relate to the changes in absorption and colour. It was also reported that Bc may be regenerated after heat treatment in the presence of ascorbic acid. This regeneration depends on the pH and at a higher pH, the regeneration of Bc is increased [7].

It was found in the preliminary study that both beetroot and Robusta are red/purple in colour, which means that colour (an intrinsic factor) may have more influence on betalain yield, stability, and food application than plant source (beetroot or cactus pear). Moreover, extrinsic factors such as extraction methods also have an impact on total betalain yield.

It was further stated by Smeriglio et al. [22] that plant complexes are more important than isolated molecules for nutraceutical and pharmaceutical uses. 

### 2.2. TSS (°Brix)

°Brix is an indication of the sugar content in fruit and has a positive influence on the sweet taste and sensory acceptability of food products [31]. Table 2 presents an analysis of °Brix, conducted on freshly-centrifuged cactus pear pulp; the °Brix extract had an impact on the final taste of the food products. Monterey (13.9%), with the highest numerical value, does not only impart colour to food products, but a sweet sensory effect was detected as well. Robusta (10.8%) and beetroot (5.5%) which had the lowest °Brix levels, both do not taste sweet and had low °Brix levels. The TSS (°Brix) were determined in order to calculate the reference value that will be used in the decision tree in the classification process of the extracts.

### 2.3. TLC

**Figure 1:**
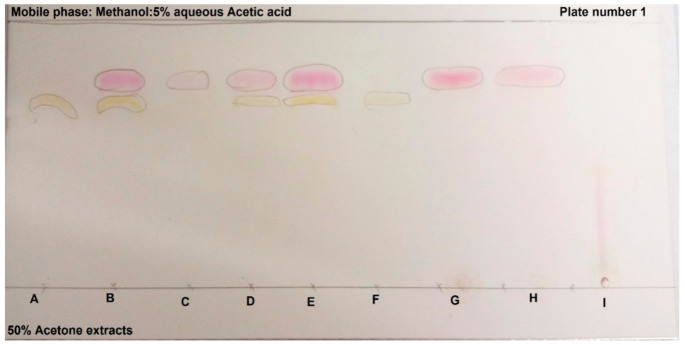
TLC plate visualized under short and long wavelength, (**A**) Ficus Indice; (**B**) Monterey; (**C**) Meyers; (**D**) Algerian; (**E**) Robusta; (**F**) Gymno carpo; (**G**) Beetroot 1; (**H**) Beetroot 2; (**I**) Amaranth.

**Figure 2:**
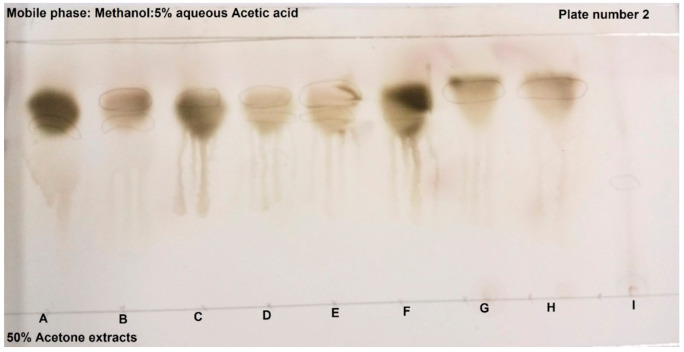
TLC was sprayed with vanillin reagent mixture, (**A**) Ficus Indice; (**B**) Monterey; (**C**) Meyers; (**D**) Algerian; (**E**) Robusta; (**F**) Gymno carpo; (**G**) Beetroot 1; (**H**) Beetroot 2; (**I**) Amaranth.

TLC results are indicated to confirm the presence and classification of true betalains as well as to indicate the presence of Bc and Bx. The two figures (Figure 1 and Figure 2) display bands on the TLC plates from amaranth, cactus pear, as well as beetroot. From Figure 1 it can be seen that the two purple (Monterey and Robusta), as well as the red Algerian cultivar, showed similar patterns with the most prominent red (Bc) and yellow (Bx) bands. The other red cultivar, Meyers, showed only Bc (red) bands, while the two orange cultivars had only the Bx pigments. Interestingly, the two beetroot samples (purple) only had the Bc values, while the purple amaranth showed a different (straight, wispy, and oval) pattern where trails of pink/red and yellow were very light. It is interesting to note the difference between beetroot with (sample G) and beetroot without AA (sample H). The cause of this difference was explained in Section 2.1.

Figure 2 indicates the results of the TLC plates treated with vanillin. Vanillin binds to amines (which are the basic structure of betalains), to verify if the observed bands are true betalains. The band patterns of samples A and F (the two orange cultivars) were similar, samples B and E (the two purple cultivars), as well as samples G and H (the two beetroot cultivars) were also similar. Of interest were samples C and D, the two red/pink cultivars that showed different patterns, as well as the similarity between samples D and E, the red/pink and purple cultivars. The two orange cactus pear cultivars, Ficus Indice, as well as the pink/red Meyers showed the most prominent bands. Gymno Carpo only showed bands of betaxanthins. The results in Table 3, show that Bc (red) pigments were found in the purple pigment sources Monterey, Robusta, both beetroot samples, as well as in the red Algerian and Meyers. However, Bc pigments were absent in the two orange cultivars, Ficus indica and Gymno carpo, as well as Amaranth. Yellow (Bx) pigments were found in the orange Ficus Indice and Gymno Carpo, red Algerian, as well as the purple Monterey and Robusta cactus pear cultivars.

### 2.4. Classification of Colourants According to the Decision Tree

**Figure 3:**
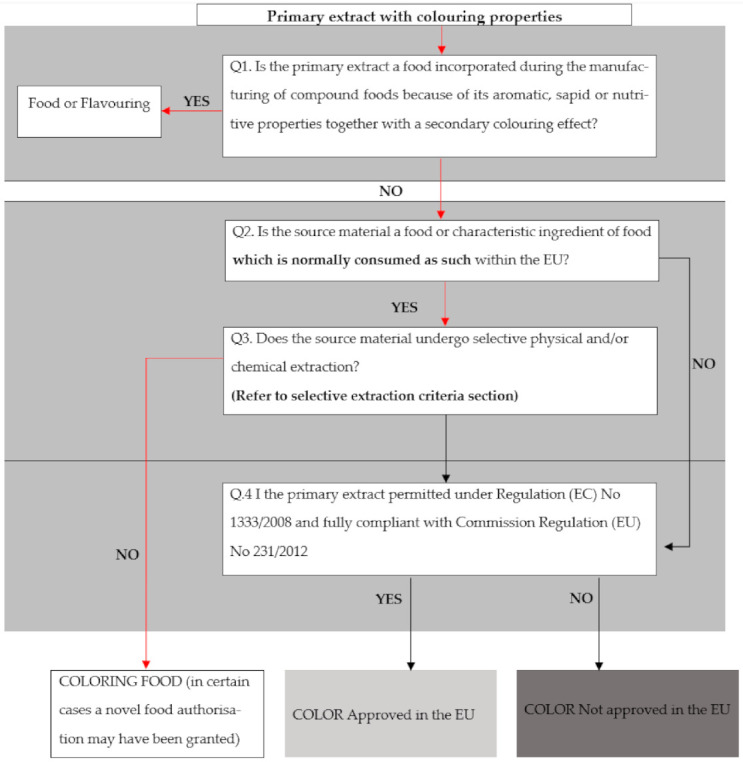
Decision tree for the classification of colourants.

Figure 3 shows the decision tree which was designed according to the EU guidelines; the responses (yes or no) are related to the extracted pigments of the current research, and the pathway followed to answer all the questions are indicated in red.

According to the pathway followed in the decision tree, by responding to the questions asked on the diagram, the current betalain extracts can, therefore, be classified as a colouring food. Lehto et al. [19] stated that vegetable and fruit juice that are classified as colouring foods, are not listed as colourants with EU numbers, and are permitted for use in the EU and USA. According to Stich [32], colouring foods are food with colouring properties and are extracted with the intent to add colour to foodstuffs. These colouring foods include carrots, spinach, and spirulina. Moreover, these colouring foods are described as juices, edible, non-selective, not standardized, and have a low colour intensity.

Firstly, the threshold should be above 6.6 to be regarded as non-selective colourants that are colouring foods. Reported TSS values (°Brix) were used as nutritive values. Both the purple cactus pear cultivars and beetroot are above that prerequisite value (as indicated Table 4). Therefore, it can be deduced that beetroot and Robusta can be viewed and classified as colouring foods.

Secondly, the extracted pigments should possess primary or secondary colouring effects. For this research, the extracts are primarily used for colouring purposes.

Thirdly, consideration is on whether the pigments are concentrated, such as freeze-dried. Most extracts were not concentrated. The concentration of pigments mainly took place during the analysis of TLC and antioxidants.

Finally, the selectivity or non-selectivity of pigments is dependent on the ratio of pigments to nutritive or aromatic constituents [32].

## 3. Materials and Methods

### 3.1. Sample Collection

#### 3.1.1. Preliminary Extraction and Antioxidant Analysis

During a preliminary study, different extraction methods (physical, chemical, and heat treatments) were tested on beetroot [20,21]. The main reason being the readily availability of beetroot throughout all seasons. The physical extraction tools (magnetic stirrer, ultrasonic bath, and liquidizer) all gave different betalain results upon extraction. The contributing factors to the extraction tool results were, among others, the particle sizes of the samples upon extraction. The liquidizer gave the highest betalain yields because it transformed samples into very fine particles with an increased total surface area. Therefore, size of the extracted particles had a significant effect on the yield of betalains: the smaller particle sizes produced higher betalain yields. For chemical extraction, water, 10–50% ethanol (EtOH) and 10–50% methanol (MeOH) were evaluated with the best extraction being at 50% EtOH. Heat treatments included a waterbath at different temperatures (5, 25, and 40 °C), a microwave oven (1000 W) at medium setting (50%) for 10, 20, and 30 s, as well as stove-top heating for 60, 120, and 180 s. The highest betalain contents were obtained with microwave extraction with or without ascorbic acid (AA) for 10 s. However, adding AA to samples altered the original colour of betalain pigments to a lighter shade of red. Highest extraction efficiency (highest total betalain and Bc and Bx contents) was obtained with liquidizing the samples in water and a heat treatment of 10 s without AA. It was therefore decided to omit the AA addition in further analysis. It should be mentioned here that the study focused on increasing the yield and concentration of betalains while at the same time taking into consideration the economic advantage of the improved extraction method.

#### 3.1.2. Beetroot

**Figure 4:**
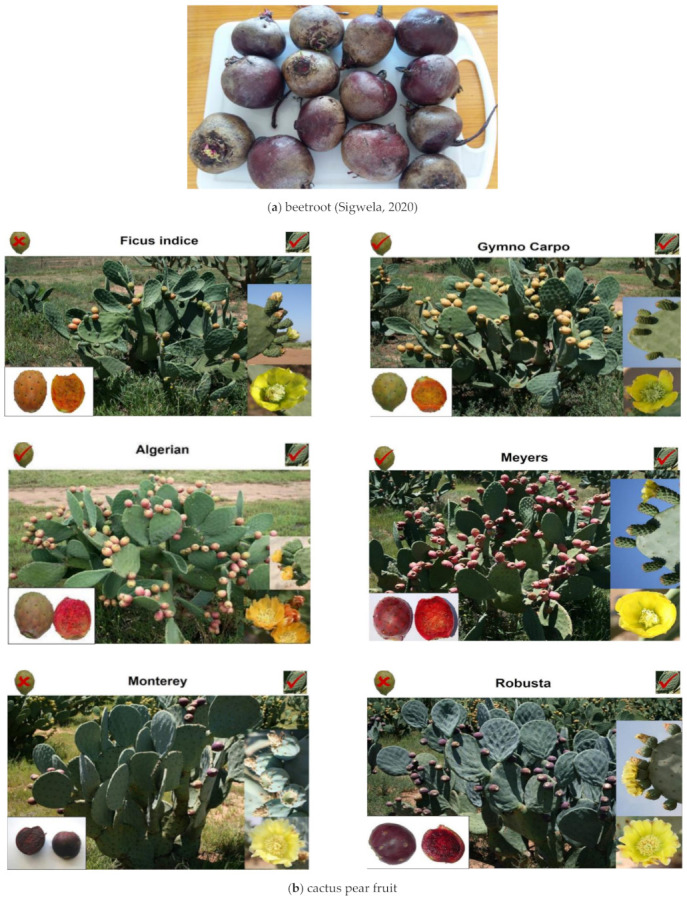
Beetroot samples (**a**) and cactus pear cultivars with different fruit colours (**b**) (courtesy of Dr. H. Fouche).

Beetroot (*Beta vulgaris* L.), Detroit Red cultivar was bought in January 2017, from a local supermarket in Bloemfontein, South Africa (Figure 4). Three batches of three roots per batch were extracted and subsequently analysed. Beetroot samples were diced into 0.1 × 0.1 cm^3^ sample size and divided into two different groups. For beetroot 1 samples, 100 g of beetroot was used and extracted with 100 mL dH_2_O, without the addition of AA. Beetroot 2 samples were prepared by adding 5 mL of a 5% AA solution to 100 g of diced beetroot samples and adding 100 mL dH_2_O. Both beetroot 1 and 2 samples were placed in microwave-safe dishes and heated for 10 s at medium (50%) heat setting, using a Defy microwave (1000 W). Addition of ascorbic acid (AA) was done in order to stabilize the betacyanin (Bc) molecules by a slightly acidic pH and to inhibit oxidation of Bc by polyphenol oxidase and tyrosinase activity when betaxanthin (Bx) is present [26]. AA was reported to prevent quick degradation of pigments as well as to cause rapid extraction of the pigments. For betalain extraction, the samples were liquidized and homogenized with a stick blender [33] and centrifuged at 9000 rpm at 4 °C for 15 min.

The extracted samples were immediately placed in sealable containers and cooled in ice-cold water to stop further cooking [34]. Some of the samples were used in their liquid state (TSS tests and colour classification), and the rest were freeze-dried. The samples that were freeze-dried were frozen at −18 °C for 24 h and subsequently freeze-dried at −20 °C for 72 h using the Labconco FreeZone^®^ Cascade Benchtop Freeze Dry System, and crushed to obtain the pigment powder.

#### 3.1.3. Cactus Pear

It was previously determined [29] that green fruit did not contain significant amounts of betalains and were therefore omitted in the current study. Thus, six cactus pear fruits from three different coloured fruit cultivars; the orange (1) Ficus Indice and (2) Gymno Carpo; pink/red fruit cultivars; (3) Algerian and (4) Meyers, as well as the purple; (5) Monterey and (6) Robusta were harvested at 50% colour-break stage (Figure 4). Three batches of each cultivar, consisting of six fruits each, were analysed. Cactus pear fruits were kept in sealable plastic bags and stored at −20 °C until used (less than a month). The peeled cactus pear fruit was liquidized and passed through a 5 mm mash-size sieve to remove the seeds. For extraction, 100 g of sample was extracted using 100 mL dH_2_O; and heated in the microwave for 10 s. The samples were liquidized using a Safeway stick blender. The liquid pulp was strained through a 5 mm mash-size sieve, centrifuged and the supernatant frozen at −18 °C for 24 h and subsequently freeze-dried and crushed to obtain the pigment powder used in tests.

#### 3.1.4. Amaranth

The purple amaranth (*Amaranthus tricolour* L.) flower leaves were picked in the summer of 2017, from the ARC Vegetable and Ornamental Institute in Roodeplaat, Pretoria, Gauteng Province, South Africa. Upon picking, the leaves were placed in sealable plastic bags and frozen at −20 °C until used. Three batches of leaves were picked and used. One hundred grams of leaves were placed in 100 mL dH_2_O and heated in the microwave for 10 s. The samples were liquidized using a Safeway stick blender. The liquid pulp was centrifuged and the supernatant frozen at −18 °C for 24 h and subsequently freeze-dried and crushed to obtain the pigment powder used in tests.

### 3.2. Methods Overview

**Figure 5:**
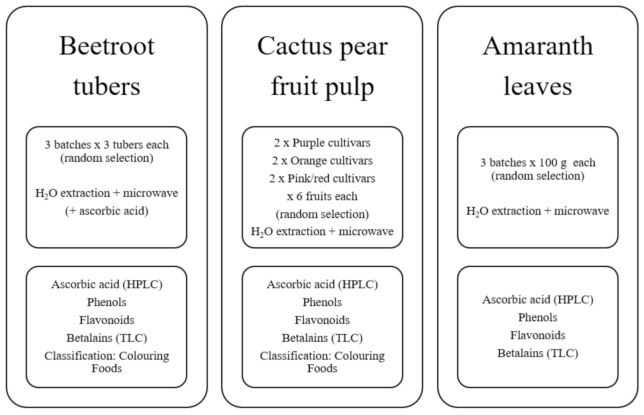
Experimental lay-out.

The experimental lay-out included three batches of randomly selected samples, either from the experimental orchards (cactus pear and amaranth) or from a local store (bags of beetroot). Each beetroot batch consisted of three tubers; each batch of fruit from the six cactus pear cultivars consisted of six fruits randomly picked from two replications and each batch of amaranth consisted of approximately 100 g of leaves, randomly selected from the orchard (Figure 5).

In the preliminary study [20,21], the antioxidant activity as well as the presence of flavonoids (phenols) of beetroot and purple, pink/red and orange cactus pears were determined by thin layer chromatography (TLC) using DPPH (1,1-diphenyl-2-picrylhydrazyl) and FeCl_3_ respectively. Purple tissues (beetroot and purple cactus pear) had the highest antioxidant activity, while purple and orange tissues (beetroot and purple as well as orange cactus pears) had similar contents of flavonoids. These results necessitated the evaluation of individual antioxidants.

#### 3.2.1. Antioxidants

Antioxidant tests included ascorbic acid (vitamin C), total phenols and flavonoid contents, done by HPLC and spectrophotometry, respectively, on freeze-dried extracts of beetroot 1 and 2, cactus pear (orange Ficus Indice and Gymno Carpo, pink/red Algerian and Meyers, purple Robusta and Monterey), as well as amaranth.

#### 3.2.2. Total Phenols

The phenolic content of beetroot 1 and 2, six cactus pear cultivars, and amaranth was determined through a method established by Makkar [34] and modified by Fawole et al. [35]. In a test tube, 50 µL betalain extract was mixed with 450 µL of 50% methanol followed by the addition of 500 µL Folin–Ciocalteu reagent and then sodium carbonate (2%) solution after 2 min. The mixture was vortexed, and absorbance read at 725 nm using a UV–visible spectrophotometer (Thermo Scientific Technologies, Madison, Wisconsin). Gallic acid standard curve (0.02–0.10 mg/mL) was used, and total phenols were expressed as mg Gallic acid equivalents GAE/100 g).

#### 3.2.3. Flavonoids

The flavonoid content of beetroot 1 and 2, six cactus pear cultivars and amaranth was determined according to an established method by Zhishen et al. [36]. One millilitre of each extract was extracted with 50% methanol (10 mL) and vortexed for 30 s. The mixture was sonicated in an ultrasonic bath for 10 min and centrifuged. Distilled water (1.2 mL) was added to 250 µL of extracted betalain samples and then followed by 75 µL of 5% sodium nitrite. After 5 min, freshly prepared 10% aluminium chloride (150 µL) was added to the mixture, followed by the addition of 500 µL sodium hydroxide after another 5 min, and 775 µL distilled water bringing the final volume to 3 mL. The mixture was vortexed, and absorbance was immediately read using a spectrophotometer at 510 nm.

The calibration curve was plotted using catechin (St. Louis, MO, USA) as a standard, and each sample was tested in triplicate. The content was expressed in mg catechin equivalents (CE) per gram [37].

#### 3.2.4. Ascorbic Acid

The ascorbic acid content of beetroot, six cactus pear cultivars and amaranth was determined according to an established method by Moyo et al. [38], where 1 g of each sample was weighed in a tube. Thereafter, 10 mL of 50% metaphosphoric acid was added to the tube. The samples were then sonicated in an ice-cold water bath for 15 min, centrifuged at 9000 rpm at 4 °C for 15 min and filtered through Whatman™ filter paper. The tests were done in the Prominence-i HPLC-PDA model system that has a sample cooler LC–2030C (Shimadzu, Kyoto, Japan).

The samples were chromatographically separated with a C18 Luna^®^ column (150 × 4.6 mm, 5 μL), which was kept at 25 °C. Next, an isocratic mobile phase was used, comprising of water (99): acetonitrile (0.9): formic acid (0.1), at a flow rate of 1 mL per min. A photodiode array detector (HPLC-PDA) was used. The samples were quantified on the calibration curve, which was plotted with l-ascorbic acid.

#### 3.2.5. Total Soluble Solids (TSS) (°Brix)

TSS (°Brix) sugars were conducted on beetroot 1 and 2 and six cactus pear cultivars, by using an Atago hand-held refractometer. This was done through squirting two drops of centrifuged samples onto the refractometer, which was held against light and the readings were recorded. The test was done in triplicate for each sample; the mean values were recorded and analysed.

#### 3.2.6. Thin Layer Chromatography (TLC)

The TLC of beetroot 1 and 2, amaranth and six cactus pear cultivars were done according to the method used by Fawole et al. [35], where 10 µL of the sample was spotted on TLC plates (Silica gel 60 F254, Merck, Germany). Two plates were developed using methanol and 5% aqueous acetic acid in 50% acetone. The first plate was visualized under short and long wavelengths, whereas the second plate was dipped in a vanillin reagent mixture, which was reported to bind to amines. After development, the plates were dried and viewed under UV light (254, 366 nm low nm for Bx and high nm for Bc). If the sample tests positive for betalains, it verifies that the colouring components of the samples are not from anthocyanins. Quantification of TLC *Rf* values (retention factor) was calculated for the TLC samples, by using a ruler to measure the distance between the solvent and solute, the calculation is shown below:

$$Rf\;\mathrm{value}\;\;=\;\frac{\mathrm{distance}\;\mathrm{travelled}\;\mathrm{by}\;\mathrm{solute}\;\left(\mathrm{cm}\right)}{\mathrm{distance}\;\mathrm{travelled}\;\mathrm{by}\;\mathrm{solvent}\;\left(\mathrm{cm}\right)}$$

#### 3.2.7. Classification of Colouring Foods

The classification of colourants was determined according to the decision tree in Figure 1 and the calculation of the threshold value [32]. There is a classification which standardises the naturalness of food, as not all natural colourants are edible. With that, the EU guidelines define selective and non-selective, giving guiding principles through the calculation of threshold values and enrichment factors [18].

The extracts can be referred to as vegetable or fruit juice if they can be consumed as food [39]. The ratio of pigments to nutritive ingredients determines whether colourants can be classified as Colouring Foods. Threshold values for the selective value should be above 6.6; it is the borderline that differentiates between selective and non-selective extraction.

The European Commission (EC) [40] established guidelines which determine whether colourants fall under the group of natural colourants or colouring foods. The guidelines include the calculation of the enrichment factor and following the guidelines of the decision tree.

Calculation of the enrichment factor:

$$Fn\;\left(\mathrm{enrichment}\;\mathrm{factor}\right)\;=\;\frac{\frac{Cp\;\left(\mathrm{pigment}\;\mathrm{in}\;\mathrm{extract}\right)}{Np\;\left(\mathrm{nutritive}\;\mathrm{content}\;\mathrm{in}\;\mathrm{extract}\right)}}{\frac{Cs\;\left(\mathrm{pigment}\;\mathrm{in}\;\mathrm{source}\right)}{Ns\;\left(\mathrm{nutritive}\;\mathrm{content}\;\mathrm{in}\;\mathrm{source}\right)}}$$

Decision tree.

The decision tree is a step-by-step process which determines whether an extract is an additive, whether the EU approves it and if it can be classified as a Colouring Food. The decision tree is elaborated on in Figure 3 [32].

### 3.3. Statistical Analysis

Antioxidants (ascorbic acid, total phenols, and flavonols) were measured on three individual samples from six cactus pear cultivars, one amaranth cultivar, and one beetroot cultivar. The effect of cultivar on antioxidant content was analysed with one-way analyses of variance (ANOVA) procedure [41]. Means were compared with the Tukey–Kramer multiple comparison test at α = 0.05 [41]. The results for all other analyses are presented as the means of triplicate analyses on three individual samples.

## 4. Conclusions

Antioxidant presence in pigment sources provides healthful benefits to consumers.

The aim of this study was to determine the biological antioxidant properties of betalain pigments from the pulp of six cactus pear cultivars, with different fruit colours, beetroot tubers, and amaranth leaves. The phenolic, flavonoid and ascorbic acid content in beetroot, amaranth, and cactus pear tissues were determined and compared. Thin layer chromatography (TLC) was applied to test and confirm Bc and Bx presence and content. Classification of betalain extracts was done according to the EU standards of colouring foods (Code of Federal Regulations/Food Additives Regulation of the EU) using pigment and TSS (°Brix) values of the extracts. In the property analysis of beetroot, six cactus pear cultivars, and amaranth, the betalain extracts had varying contents of phenols, flavonoids, and ascorbic acid.

Antioxidant contents were higher in amaranth, and beetroot that was heated with AA. The antioxidants in amaranth were significantly higher than all the other samples, with 34.91 mgGAE/g in phenols, 5.07 mg CE/g in flavonols, and 71.71 mg/100 g ascorbic acid. Beetroot samples that were heated without AA had the lowest numerical value for all antioxidants. Betalain extracts from amaranth contained the highest betalains content compared to betalain extracts from beetroot and cactus pear. These results show that betalain presence is dependent on extraction method and betalain source. Results further showed that Robusta (purple), Monterey (purple), and Gymno Carpo (orange) fruit had the highest betacyanin and betaxanthin content in the cactus pear cultivars. The extracts can be classified as colouring foods. The manuscript further reports the watery extraction of betalains from natural plant sources by means of a fast, cheap, and green microwave extraction method as well as its subsequent classification as Colouring Foods and as bioactive antioxidant-rich sources.

## Figures and Tables

**Table 1:** Content of antioxidants in different cultivars.

Cultivar	Total Phenols (mg GAE/g)	Total Flavonoids (mg CE/g)	Ascorbic Acid (mg/100 g)
Ficus indica	5.35 ^b^ ± 0.30	0.63 ^ab^ ± 0.02	12.35 ^d^ ± 0.08
Gymno carpo	7.00 ^b^ ± 0.11	3.15 ^f^ ± 0.15	11.10 ^a^ ± 0.19
Algerian	5.26 ^b^ ± 0.04	0.62 ^ab^ ± 0.01	12.17 ^cd^ ± 0.07
Meyers	5.38 ^b^ ± 0.11	0.69 ^b^ ± 0.04	12.30 ^cd^ ± 0.25
Monterey	5.96 ^b^ ± 0.20	1.39 ^c^ ± 0.07	11.71 ^bc^ ± 0.03
Robusta	6.27 ^b^ ± 0.55	1.62 ^d^ ± 0.03	11.45 ^ab^ ± 0.14
Beetroot 1	3.18 ^a^ ± 0.03	0.49 ^a^ ± 0.07	11.11 ^ab^ ± 0.13
Beetroot 2	29.86 ^c^ ± 1.40	1.84 ^e^ ± 0.04	12.09 ^cd^ ± 0.05
Amaranth	34.91 ^d^ ± 1.35	5.07 ^g^ ± 0.05	71.71 ^e^ ± 0.51
Significance level	*p* < 0.001	*p* < 0.001	*p* < 0.001

Means with different superscripts in the same column differ significantly; Beetroot 1 (Microwave heated + no AA); Beetroot 2 (Microwave heated + AA).

**Table 2:** °Brix for beetroot 1 and six cactus pear cultivars.

Cultivar	Sugar (Glc and Fru) (%)	Standard Deviation
Ficus Indice	13.3	1.1
Gymno Carpo	12.2	1.0
Algerian	13.1	0.9
Meyers	11.9	1.4
Monterey	13.9	1.0
Robusta	10.8	0.7
Beetroot	5.5	1.0

Mean values of three replications.

**Table 3:** *Rf* values calculated for TLC samples with (Figure 1) and without vanillin (Figure 2).

Sample		Without Vanillin		With Vanillin	
TLC Code	Cultivar	*Rf*	*Rf*	*Rf*	*Rf*
A	Ficus Indice	NM	0.70	NM	0.75
B	Monterey	0.78	0.70	0.77	0.71
C	Meyers	0.77	NM	0.76	NM
D	Algerian	0.70	0.78	0.77	0.70
E	Robusta	0.80	0.72	0.80	0.71
F	Gymno Carpo	NM	0.72	NM	0.73
G	Beetroot 1	0.80	NM	0.80	NM
H	Beetroot 2	0.80	NM	0.80	NM
I	Amaranth	NM	NM	NM	NM

NM: Not Measured; Beetroot 1 (Microwave heated + no AA); Beetroot 2 (Microwave heated + AA).

**Table 4:** Colouring food classification for beetroot and purple cactus pear cultivar.

	Beetroot	Purple Cactus Pear Fruit (Robusta)	Calculated/Obtained from
Cp (pigment in extract) = Bc (mg/g)	302.04	669.93	Bc and Bx values obtained in preliminary study (results not shown) of watery extract
Np (nutritive content in source) = TSS = °Brix	5.50	10.80	TSS (°Brix determined and indicated in Table 2 (extract)
Cs (pigment in source) = Bc (mg/g)	54.00	77.47	Bc values for whole fruit/root obtained from literature
Ns (nutritive content in source) = TSS = °Brix	9.40	9.50	TSS (°Brix) obtained from literature for whole fruit/root
Threshold	7.9	8.9	>6.6

## Sample Availability

Samples of the compounds are available from the authors.

## References

[1] Gengatharan A., Dykes G.A., Choo W.S. (2015). Betalains: Natural plant pigments with potential application in functional foods. LWT Food Sci. Technol..

[2] Del Socorro Santos Díaz M., Barba de la Rosa A.P., Héliès-Toussaint C., Guéraud F., Nègre-Salvayre A. (2017). *Opuntia* spp.: Characterization and benefits in chronic diseases. Oxid. Med. Cell. Long..

[3] Neha P., Jain S.K., Jain N.K., Jain H.K., Mittal H.K. (2018). Chemical and functional properties of Beetroot (*Beta vulgaris* L.) for product development. Int. J. Comm. Syst..

[4] Khan M.I. (2016). Plant betalains: Safety, antioxidant activity, clinical efficacy, and bioavailability. Compreh. Rev. Food Sci. Food Saf..

[5] Antigo J.L.D., Bergamasco R.D.C., Madrona G.S. (2018). Effect of pH on the stability of red beet extract (*Beta vulgaris* L.) microcapsules produced by spray drying or freeze drying. Food Sci. Technol..

[6] Prieto-Santiago V., Cavia M.M., Alonso-Torre S.R., Carrillo C. (2020). Relationship between color and betalain content in different thermally treated beetroot products. J. Food Sci. Technol..

[7] Fu Y., Shi J., Xie S.Y., Zhang T.Y., Soladoye O.P., Aluko R.E. (2020). Red beetroot betalains: Perspectives on extraction, processing, and potential health benefits. J. Agric. Food Chem..

[8] Solovchenko A., Yahia E.M., Chen C., Yahia E.M. (2019). Pigments. Postharvest Physiology and Biochemistry of Fruits and Vegetables.

[9] Skalicky M., Kubes J., Shokoofeh H., Tahjib-Ul-Arif M., Vachova P., Hejnak V. (2020). Betacyanins and Betaxanthins in cultivated varieties of *Beta vulgaris* L. compared to Weed Beets. Molecules.

[10] Herbach K.M., Stintzing F.C., Carle R. (2006). Betalain stability and degradation—Structural and chromatic aspects. J. Food Sci..

[11] Kataoka H. (2019). Pharmaceutical analysis/Sample Preparation. Reference module in Chemistry, Molecular Sciences and Chemical Engineering. Encyclopedia of Analytical Science.

[12] Gomez L., Tiwari B., Garcia-Vaquero M. (2020). Emerging extraction techniques: Microwave-assisted extraction. Sustainable Seaweed Technologies.

[13] Zin M.M., Bethel C.A., Bánvölgyi S. (2020). Recovery of phytochemicals via electromagnetic irradiation (Microwave-Assisted-Extraction): Betalain and phenolic compounds in perspective. Foods.

[14] Pasqueta V., Cherouvrier J.-R., Firas F., Picot L. (2011). Study on the microalgal pigments extraction process: Performance of microwave assisted extraction. Process. Biochem..

[15] Maqsood U.R., Fazlullah A.K., Kamal N. (2020). Introduction to natural products analysis. Recent Advances in Natural Products Analysis.

[16] Singh A., Singh K., Kumar H., Abhay R., Pandey K. (2020). Analysis of chlorophylls. Recent Advances in Natural Products Analysis.

[17] Cardoso-Ugarte G.A., Sosa-Morales M.E., Ballard T., Liceaga A., Martin-Gonzalez M.F.S. (2014). Microwave-assisted extraction of betalains from red beet (*Beta vulgaris*). Food Sci. Technol..

[18] Stich E. (2016). Handbook on Natural Pigments in Food and Beverages.

[19] Lehto S., Buchweitz M., Klimm A., Straßburger R., Bechtold C., Ulberth F. (2017). Comparison of food colour regulations in the EU and the US: A review of current provisions. Food Addit. Contam. Part A.

[20] Sigwela V. (2020). Extraction, Characterization and Application of Betalains from Cactus Pear, Beetroot and Amaranth. Master’s Thesis.

[21] Sigwela V.N., de Wit M., Amoo S., Hugo A., du Toit A. Extraction, Characterization and Application of Betalains from Beetroot, Cactus Pear and Amaranth for Food Safety. Presented at the Second International Congress on Food Safety and Security.

[22] Smeriglio A., Bonasera S., Germanò M.P., D’Angelo V., Barreca D., Denaro M., & Trombetta D. (2019). *Opuntia ficus-indica* (L.) Mill. fruit as source of betalains with antioxidant, cytoprotective, and anti-angiogenic properties. Phytother. Res..

[23] Koss-Mikołajczyk I., Kusznierewicz B., Wiczkowski W., Sawicki T., Bartoszek A. (2019). The comparison of betalain composition and chosen biological activities for differently pigmented prickly pear (*Opuntia ficus-indica*) and beetroot (*Beta vulgaris*) varieties. Int. J. Food Sci. Nutr..

[24] Albano C., Negro C., Tommasi N., Gerardi C., Mita G., Miceli A., De Bellis L., Blando F. (2015). Betalains, phenols and antioxidant capacity in cactus pear [*Opuntia ficus-indica* (L.) Mill.] fruits from Apulia (South Italy) genotypes. Antioxidants.

[25] Nowak D., Goslinski N., Wojtowicz M., Przygonski K. (2018). Antioxidant properties of phenolic compounds of vitamin C-rich fruit juices. J. Food Sci..

[26] Kolniak-Ostek J., Oszmianski J., Wojdylo A. (2013). Effect of L-ascorbic acid addition on quality, phenolic compounds and antioxidant activity of cloudy apple juices. Eur. Food Res. Technol..

[27] Skopińska A., Szot D., Starzak K., Mizrahib Y., Wybraniec S. (2015). The effect of ascorbic acid supplementation on betacyanin stability in purple pitaya (*Hylocereus polyrhizus*) juice. Chall. Mod. Technol..

[28] Du Toit A. (2013). Antioxidant Content and Potential of Fresh and Processed Cladodes and Fruit from Different Coloured Cactus Pear (*Opuntia ficus-indica*) Cultivars. Master’s Thesis.

[29] Du Toit A., De Wit M., Osthoff G., Hugo A. (2018). Relationship and correlation between antioxidant content and capacity, processing method and fruit colour of cactus pear fruit. Food Bioprocess. Technol..

[30] Ravichandran K., Saw N.M.M.T., Mohdaly A.A.A., Gabr A.M.M., Kastell H.R., Cai Z., Knorr D., Smetanska I. (2013). Impact of processing of red beet on betalain content and antioxidant activity. Food Res. Int..

[31] Kgatla T.E., Howard S.S., Hiss D.C. (2011). Colour stability of wild cactus pear juice. Int. J. Nutr. Food Eng..

[32] Reinhart A. (2014). Colouring Foods versus Food Colours: Guidance notes on the classification of food extracts with colouring properties. Eur. Food Feed Law Rev..

[33] Stintzing F.C., Herbach K.M., Mosshammer M.R., Carle R., Yi W., Sellappan S., Akoh C.C., Bunch R., Felker P. (2005). Colour, betalain pattern, and antioxidant properties of cactus pear (*Opuntia* spp.) clones. J. Agri. Food Chem..

[34] Makkar H.P.S. (1999). Quantification of Tannins in Tree Foliage: A Laboratory Manual for the FAO/IAEA Co-Ordinated Research Project on Use of Nuclear and Related Techniques to Develop Simple Tannin Assays for Predicting and Improving the Safety and Efficiency of Feeding Ruminants on the Tanniferous Tree Foliage.

[35] Fawole O.A., Ndhlala A.R., Amoo S.O., Finnie J.F., Van Staden J. (2009). Anti-inflammatory and phytochemical properties of twelve medicinal plants used for treating gastro-intestinal ailments in South Africa. J. Ethnopharmacol..

[36] Zhishen J., Mengcheng T., Jianming W. (1999). The determination of flavonoid contents in mulberry and their scavenging effects on superoxide radicals. Food Chem..

[37] Amoo S.O., Van Staden J. (2012). Influence of plant growth regulators on shoot proliferation and secondary metabolite production in micropropagated Huernia hystrix. Plant Cell Tissue Organ Cult. (PCTOC).

[38] Moyo M., Amoo S.O., Aremu A.O., Gruz J., Šubrtová M., Jarošová M., Tarkowski P., Doležal K. (2018). Determination of mineral constituents, phytochemicals and antioxidant qualities of *Cleome gynandra*, compared to *Brassica oleracea* and *Beta vulgaris*. Front. Chem..

[39] FDA (Food and Drug Administration), U.S. Department of Health and Human Services Food and Drug Administration Center for Food Safety and Applied Nutrition (2017). Juice HACCP and the FDA Food Safety Modernization Act: Guidance for Industry.

[40] European Union (2008). Commission Directive 2008/128/EC of 22 December 2008 Laying down Specific Purity Criteria Concerning Colours for Use in Foodstuffs (Codified Version). Off. J. Eur. Union..

[41] (2016). NCCS 11 Statistical Software.

